# Synthesis and Biological Evaluation of Novel Aromatic Imide-Polyamine Conjugates

**DOI:** 10.3390/molecules21121637

**Published:** 2016-11-30

**Authors:** Ming Li, Yuxia Wang, Jianying Zhang, Songqiang Xie, Chaojie Wang, Yingliang Wu

**Affiliations:** 1 Department of Pharmacology, Shenyang Pharmaceutical University, Shenyang 110016, China; songxu666@163.com; 2 Pharmaceutical College, Henan University, Kaifeng 475001, China; 18237878517@163.com (J.Z.); xiesq@henu.edu.cn (S.X.); 3 College of Chemistry and Chemical Engineering, Henan University, Kaifeng 475001, China; 4 Key Laboratory of Natural Medicine and Immuno-Engineering, Kaifeng 475001, China; wcjsxq@henu.edu.cn

**Keywords:** naphthalimide, polyamine, antitumor, synthesis, amonafide

## Abstract

Three types of conjugates in which aromatic imide scaffolds were coupled to diverse amine/polyamine motifs were synthesized, and their antitumor activities were evaluated in vitro and in vivo. Results showed that the conjugate **11e** of 1,8-naphthilimide with spermine had pronounced effects on inhibiting tumor cell proliferation and inducing tumor cell apoptosis via ROS-mediated mitochondrial pathway. The in vivo assays on three H22 tumor transplant models revealed that compound **11e** exerted potent ability in preventing lung cancer metastasis and extending lifespan. Furthermore, the efficacy of **11e** in inhibiting tumor growth and improving body weight index were better than that of positive control, amonafide. Our study demonstrates that compound **11e** is a valuable lead compound for further investigation.

## 1. Introduction

**Figure 1:**
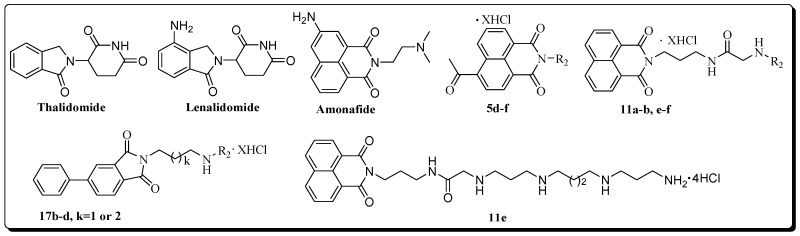
The structures of **5d**–**f**, **11a**–**b**, **e**–**f**, **17b**–**d**, thalidomide, lenalidomide and amonafide (X = 2, 3, 4; R_2_ = deprotected polyamine chain).

The development of effective anticancer agent continues to be a formidable challenge in medicine. Aromatic imides provide an excellent template for new drug development and have drawn special attention of medicinal chemists. Some phthalimides such as thalidomide and lenalidomide (Figure 1) are now in clinical use [1,2]. Thalidomide, initially introduced for treatment of multiple myeloma (MM) because of its anti-angiogenic properties, has shown remarkable activity alone and in combination with other drugs in patients across all stages of the disease. Lenalidomide has been shown to benefit patients with multiple myeloma, myelodysplastic syndromes, and lymphoma [3,4,5].

Naphthalimides are also a class of intriguing pharmacophores [6,7]. Although many naphthalimides and their synthetic analogs have reached clinical trials, most of them were abandoned because of various adverse effects. For example amonafide (Figure 1) had shown potent anticancer activity. However, due to unexpected central neurotoxicity and limited efficiency, further application in the clinical trial was abandoned [8]. In order to improve the tumor selectivity, efficiency, and safety, a broad variety of novel naphthalimide derivatives by modified amonafide at different positions have been reported [9,10].

**Figure 2:**
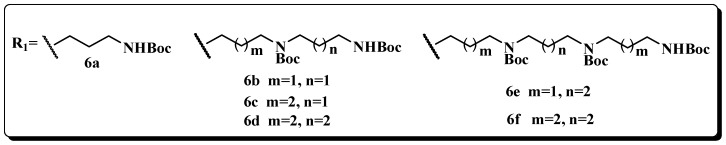
Building blocks of the polyamine skeleton.

Currently, extensive researches have emphasized the fact that polyamine conjugates acting as promising antitumor candidates are becoming increasingly important in the polyamine field [11,12,13,14,15]. F14512, an epipodophyllotoxin-spermine conjugate, is perhaps the most promising compound which is presently being evaluated in phase I trials [16,17,18,19]. Our work demonstrated that derivatives incorporating an aromatic imide skeleton or connecting to a polyamine linker such as spermidine or homospermidine, exhibit marked cell selectivity and lung cancer metastasis inhibition [2,3,4,5,6,7,8,9,10,11,12,13,14,15,16,17,18,19,20,21,22]. These findings stimulated us to search for more promising imide scaffolds suitable for polyamine modification. We report herein three kinds of aromatic imide (Figure 1), which are coupled with diverse polyamine motifs (Figure 2). In addition to the routine in vitro evaluation, in vivo experiments are designed to provide more data to justify their future drug development.

## 2. Results and Discussion

### 2.1. Chemistry

**Scheme 1:**
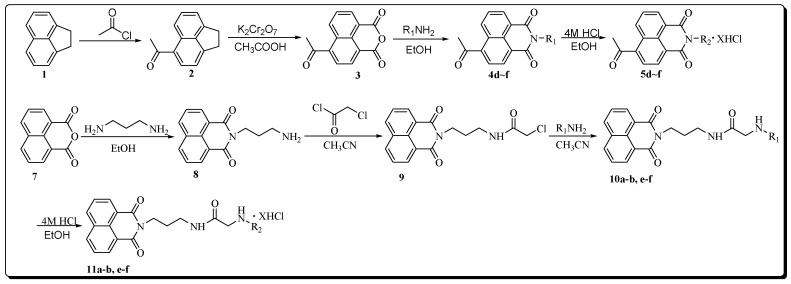
Synthetic protocol of target compounds **5d**–**f**, **11a**–**b**, **e**–**f** (X = 2, 3, 4; R_1_ = *N*-Boc protected polyamine chains; R_2_ = deprotected polyamine chains).

The acetyl-substituted naphthalimide-polyamine compounds **5d**–**f** were synthesized according to Scheme 1. Intermediates **2** and **3** were prepared by a modified previously reported procedure [23,24]. The respective *N*-Boc protected intermediates **4d**–**f** were obtained by the condensation of R_1_NH_2_ (**6**, the Boc protected polyamines were prepared by a modified previously reported procedure) [25] (as shown in Figure 2) with 4-acetyl-1,8-naphthalic anhydride (**3**). After purification by flash column chromatography, these intermediates were mixed with 4 M HCl in EtOH at room temperature to obtain the target compounds **5d**–**f** as their hydrochloride salts with a yield between 70% and 80% [22].

Compounds **11a**–**b**, **e**–**f** were synthesized following a four-step procedure as shown in Scheme 1. Compound **8** obtained by reacting 1,8-naphthalic anhydride with 1,3-propanediamine reacted with chloroacetyl chloride to give intermediate **9**. Compounds **10a**–**b**, **e**–**f** were easily formed by the reaction of **9** and R_1_NH_2_ in acetonitrile. After purification by flash column chromatography, these intermediates were mixed with 4 M HCl at room temperature to obtain the target compounds **11a**–**b**, **e**–**f** as their corresponding hydrochloride salts with a yield between 70% and 80%.

**Scheme 2:**
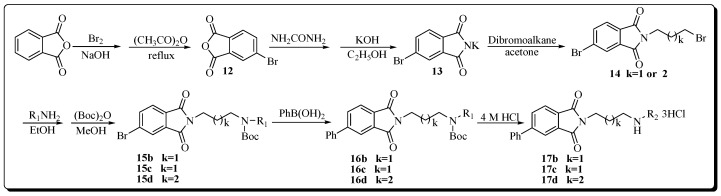
Synthetic protocol of target compounds **17b**–**d** (R_1_ = *N*-Boc protected polyamine chains; R_2_ = deprotected polyamine chains).

The synthesis of target compounds **17b**–**d** are described as in Scheme 2. Intermediates **12** and **13** were prepared by a modified previously reported procedure [26,27]. Compound **13** was N-alkylated by commercial dibromoalkane to yield intermediate **14** in good yield. Subsequently, compound **14** was condensed with corresponding amines R_1_NH_2_, after the completion of the reaction, the solvent was removed by rotary evaporation yielding a residue. This residue, without further purification, was N-protected to form **15b**–**d** using excess di-*tert*-butyl dicarbonate (Boc_2_O). Suzuki coupling of **15b**–**d** with phenylboronic acid catalyzed by PdCl_2_ gave intermediates **16b**–**d** [28]. From these compounds, Boc groups in the polyamine skeleton were subsequently removed with 4 M HCl in EtOH at room temperature to provide target compounds **17b**–**d** as hydrochloride salts [29].

### 2.2 Biological Evaluation

#### 2.2.1. Cytotoxicity in Human Tumor Cell Lines

The inhibitory activity of target compounds toward five tumor cells (HCT-116: human colorectal cancer cell line; K562: human leukemia cell line; HepG2: human hepatoma cell line; MDA-MB-231: human breast cancer cell line; QSG-7701: normal hepatocyte cell line) were measured by the traditional MTT ({3-(4,5-dimethylthiazol-2-yl)}-2,5-diphenyl-tetrazolium bromide) test. Amonafide was tested as a reference compound. Structures and biological results of target compounds are shown in Table 1. The results showed that the naphthalimide-polyamine conjugates **5d**–**f** exhibited antitumor activities as potent as previously reported naphthalimide-polyamine conjugates [2,21]. Compounds **11a**–**b**, **e**–**f** exhibited mixed results, compound **11e**, which displayed improved potency in comparison to other analogues in the series, was of comparable potency to amonafide. Surprisingly, the 5-phenylphthalimide-polyamines **17b**–**d** were not active.

#### 2.2.2. Apoptosis

**Figure 3:**
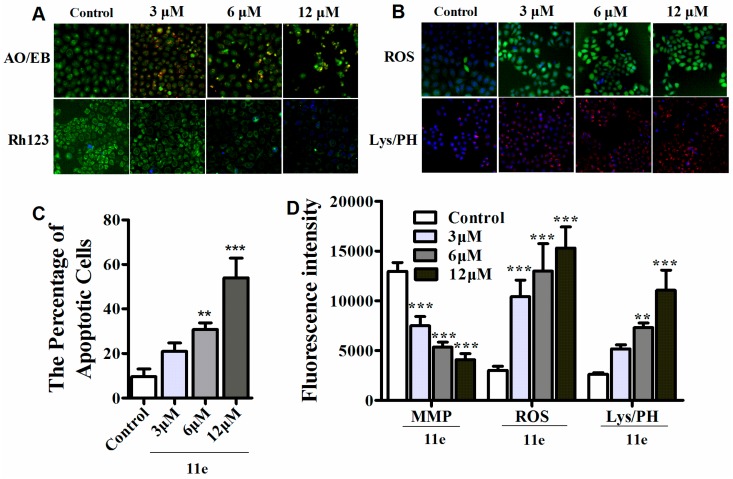
The apoptosis and MMP (mitochondrial membrane potential), ROS (reactive oxygen species) and lysosomal mass/pH (Lys/PH) were investigated in HepG2 cells in vitro. (**A**) The cellular morphous, MMP were detected with AO/EB, Rh123/Hoechst33342 staining using HCS in HepG2 cells; (**B**) ROS, lysosomal mass/pH were detected by HCS using DCFH-DA and Lyso-Tracker Red staining respectively; (**C**) **11e** induced apoptotic cells at different concentrations (3, 6, 12 μM) assessed by HCS using AO/EB staining after 48 h treatment in HepG2 cells (*n* = 3, x ± SD, ** *p* < 0.01, *** *p* < 0.001); (**D**) Impacts of **11e** at different concentrations (3, 6, 12 μM) on MMP, ROS, lysosomal mass/pH, detected by HCS using Rh123, DCFH-DA, and Lyso-Tracker Red staining respectively after 48 h treatment in HepG2 cells. Each sample is the mean of three independent experiments. (*n* = 3, x ± SD, ** *p* < 0.01, *** *p* < 0.001).

Previous studies revealed that most naphthalimide-polyamine conjugates may trigger tumor cell death via an apoptotic process [30,31]. To appraise if novel naphthalimide-polyamine conjugates induce cell apoptosis, morphological study was applied after HepG2 cells were stained with AO/EB. The percentage of apoptotic cells was calculated after observing a total of 300 cells and as shown in Figure 3A,C, obvious apoptosis induced by **11e** was observed in a dose-dependent manner [32]. To furnish more credible information about the apoptosis of HepG2 cells induced by compound **11e**, we used the high content screening (HCS) technique to detect related apoptosis indicators. Mitochondria are vital cell organelles in the process of transmitting apoptosis signals, whereas the mitochondrial membrane potential (MMP) is a critical parameter related to mitochondrial function in cells. As a MMP specific cationic fluorescent dye, Rh123 is used to measure the change of MMP, which accumulates in healthy mitochondria with intact membrane potential and is absent in the depolarized mitochondria [22]. Lysosomes, as cellular targets in cancer therapy, participate in various cell death profiles such as apoptosis and senescence [9,33,34]. Thus, the lysosomal mass/pH (Lys/pH) variation was detected by a basic fluorescent probe Lyso-Tracker Red which selectively accumulates in acidic lysosomes during cytotoxicity occurs. After treatment with compound **11e** for 48 h, the green fluorescence of Rh123 in cytoplasm was significantly decreased in a dose-dependent manner, indicating that the destroyed mitochondria lost their mitochondrial transmembrane potential (Figure 3A,D). Meanwhile, the remarkable accruement of red fluorescence in cells treated with compound **11e** implied the dose-related occurrence of lysosomes impairment (Figure 3B,D).

It is known that polyamine conjugates may produce reactive oxygen species (ROS) [22]. The elevated ROS could bring corresponding influences on MMP loss and LYS/pH destabilization, which generally activates the cell death. Therefore, the ROS generation was detected by the DCFH-DA (2′,7′-dichlorodihydrofluorescein diacetate) experiment. Indeed, the apparently increased ROS was observed in a dose-dependent pattern as shown in Figure 3B,D.

#### 2.2.3. Antitumor Activity In Vivo

**Figure 4:**
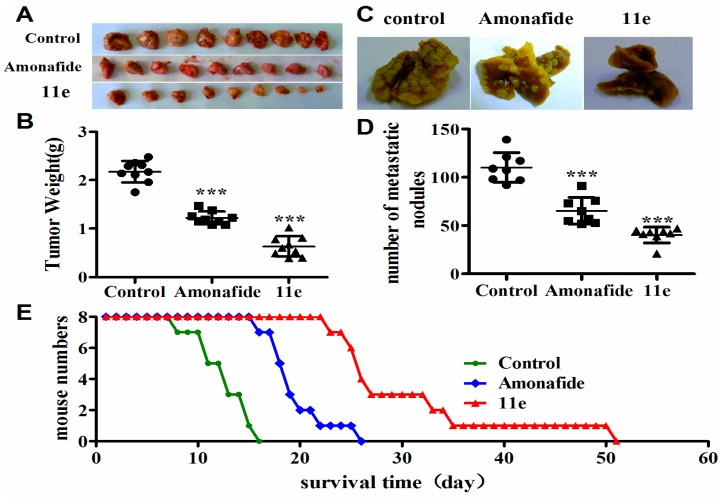
Antitumor activity of **11e** was evaluated in vivo. (**A**) Photographs of tumor obtained from each treatment group excised on day 10; (**B**) Tumor weight in subcutaneous model, compared with control: (*** *p* < 0.001, x ± SD), *n* = 9; (**C**) Lung metastasis photograph; (**D**) Lung metastasis nodus numbers for pulmonary metastasis in mice treatment with **11e**, amonafide, and normal saline. (*** *p* < 0.001, x ± SD), *n* = 8; (**E**) The changes of body weight of mice treated with **11e**, amonafide, and normal saline; (**E**) Kaplan-Meier curves showing survival of mice treated with **11e**, amonafide, and normal saline.

Several of our in vitro observations reported here demonstrate that **11e** has the ability to interfere with the proliferation of tumor cells. However, few in vivo studies provided limited messages for rational design of the conjugates in comparison to the plentiful in vitro data. Indeed, there are significant discrepancies between in vitro and in vivo experiments in many cases. In vivo trials, based on preliminary in vitro screens, provide more valuable messages for further drug development. To further evaluate the antitumor activity of **11e** in vivo, we chose three H22 (mice hepatoma cell line) tumor transplant models: solid tumor (tumor growth inhibition evaluation), ascites tumor (live time evaluation), and pulmonary metastasis tumor (tumor metastasis evaluation). As shown in Figure 4A,B and Table 2 after treatment of mice bearing hepatoma xenografts (H22 mouse hepatocellular carcinoma model) with 1 mg/kg of **11e** once every day, obvious tumor weight suppression by 70.92% was observed compared to the negative control group that treated with normal saline. Mice bearing hepatoma xenografts were also treated with 5 mg/kg of amonafide and the tumor weight suppression by 43.79% was observed compared to the negative control group. Thus, tumor growth inhibitory rates in mice treated with **11e** and amonafide were dramatically increased, and the in vitro antitumor effect of **11e** was also obviously stronger than that of amonafide.

Metastasis is the process by which tumors spread from the primary organ in which they arise to other sites in the body. In most cases, tumors that stay confined to one organ will not be fatal, tumors that have spread to other organs have a greater potential for causing harm. To test whether **11e** was effective against an established lung metastasis, we injected H22 cells intravenously into 24 Kunming mice (5 × 10^6^ cells each mouse). To ensure that all mice bore actively growing lung tumors before the drug treatment, pulmonary cancer was allowed to develop for 10 days. Mice were treated with **11e**, amonafide and normal saline for 7 consecutive days, respectively. Mice treated with **11e** displayed few metastases (62.42%), whereas all mice treated with normal saline had an extensive tumor burden in the lungs. In contrast, amonafide moderately decreased lung metastasis nodus numbers (40.7%) (Figure 4C,D; Table 3). These results supported that **11e** inhibited the tumor metastasis lighter than amonafide.

Impacts of **11e** on survival time in H22-bearing mice were evaluated by measuring the extension of the lifespan. The median survival time (MST) in control mice was (12.75 ± 2.5) days. After the **11e** or amonafide *i.v.* administrated, the life span was increased by 2.41-fold ((30.75 ± 4.4) days) or 1.53-fold (19.5 ± 3.6 days) compared with that of the control group, respectively (Figure 4E).

#### 2.2.4. Systemic Toxicity

**Figure 5:**
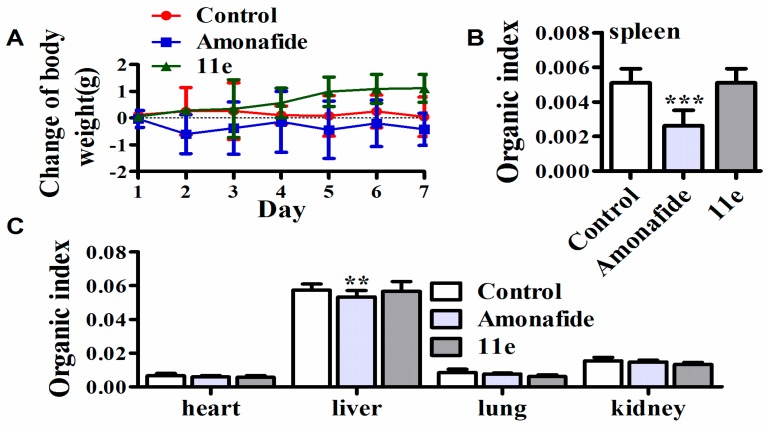
Systemic toxicity of **11e**. (**A**) The changes of body weight of mice treated with **11e**, amonafide, and normal saline; (**B**) The organ indexes of immune organ (spleen) were evaluated after treatment with **11e**, amonafide, and normal saline (*n* = 3, x ± SD *** *p* < 0.001); (**C**) The organ indexes (heart, liver, kidney and lung) were evaluated after treatment with **11e**, amonafide, and normal saline (*n* = 3, x ± s ** *p* < 0.01).

It is reported that amonafide can result in severe adverse effects and which greatly limited its clinical application [35]. In both cell and animal models, some polyamine conjugates such as F14512 has been confirmed to possess favorable cell or tissue selectivity [36], which is expected to reduce the adverse effects of parent drugs. However, there are few reports about the systemic toxicology of naphthalimide-polyamine conjugates to support the above hypothesis. Consequently, we tested the systemic toxicity of **11e** (1 mg/kg) with amonafide (5 mg/kg) in tumor-bearing mice for 7 consecutive days (intravenous injection). At the same time, the body weight was monitored to detect the dose-limited toxicity. On day 8, these mice were killed by cervical vertebra, and the organs and tissues, including samples of heart, liver, lung, kidney, spleen and thymus, were taken for the inspection of systemic toxicity. There was no weight loss in the **11e**-treated mice, on the contrary, the weight increased slightly. Whereas treatment with amonafide was associated with some degree of weight loss, indicating possibly dose-related toxicity (Figure 5A). Heart, kidney and lung had no obvious index value changes in the examination of both compounds (Figure 5C). The liver and spleen index numbers had no significant variation after **11e** treatment compared to controls, but amonafide treatment obviously decreased these index values, implying that **11e** had no influence on liver and immune function, at the same time suggesting that amonafide injured liver function and caused damage to immune function in some extent (Figure 5B,C). In short, **11e** performed better than amonafide in the experiments of the toxicological profile at the doses that exert antitumor activity in vivo.

#### 2.2.5. ADMET Prediction

The in silico prediction of physicochemical, ADME and toxicity properties of compounds **11e** was calculated using the ACD/Labs Percepta Platform 23 (License#58830) and results were compared to those obtained for amonafide. As demonstrated in Table 4, the druglikeness of compound **11e** was similar to amonafide except rotatable bonds [37,38]. Regardless of the poor human intestinal absorption (HIA), compound **11e** showed excellent solubility and general permeable based on predicted permeability across Caco-2 monolayers (Pe). The drug safety profile of these compounds was also projected using Program ACD/Percepta based on probabilistic predictors. The inhibition of hERG (human Ether-a-go-go-Related Gene) and the mutagenic profile were calculated and the results converted in the so called classification scores (Table 5). As depicted in Table 4, compound **11e** was predicted as undefined (score > 0.33 and <0.67) in HLM (human liver microsomes), hERG and mutagenic, which were similar to amonafide.

## 3. Materials and Methods

### 3.1. General Information

All solvents and reagents were purchased from the suppliers and used without further purification. All NMR spectra were recorded on an AV-400 model spectrometer (Bruker BioSpin, Zürich, Switzerland) in D_2_O or CDCl_3_, Chemical shifts for ^1^H-NMR spectra are reported in parts per million to residual solvent protons. ESI-MS spectra were recorded on an ESQUIRE-LC mass spectrometer (Agilent, Palo Alto, CA, USA). The target compounds with the purity being higher than 95% were analyzed using combustion analysis, Elemental analyses were performed on a Gmbe VarioEL elemental instrument (Elementar ,Langenselbold, Germany), and results were within 0.4% of the theoretical values.

### 3.2. General Procedure for the Synthesis of ***5d***–***f***

A mixture of 4-acetyl-1,8-naphthalic anhydride **3** (2 mmol) and polyamine **6** (2 mmol) (as shown in Figure 2) in EtOH (30 mL) was heated at refluxing temperature, and the reaction process was monitored by TLC. After completion, the reaction mixture was cooled to room temperature and concentrated under vacuum to give an oily residue, which was purified by chromatography on silica gel using petroleum ether/ethyl acetate mixture as eluent to obtain the Boc protected intermediates **4d**–**f**. The respective *N*-Boc protected intermediates **4d**–**f** (1.2 mmol) were dissolved in EtOH (20 mL) and stirred at 0 °C for 10 min. Then 4 M HCl was added dropwise at 0 °C. The reaction mixture was stirred at room temperature overnight. The solution typically gave a white solid as precipitate. The solid was filtered, washed several times with absolute ethanol, and target compounds **5d**–**f** were obtained [19].

*2-[(2-Dimethylamino)ethyl-[4-(4-aminobutylamino)butyl]-6-acetyl-1H-benz[de]isoquinoline-1,3(2H)-dione dihydrochloride* (**5d**). Yield: 83%, white solid. ^1^H-NMR (D_2_O) δ: 8.67 (d, 1H, *J* = 8.0 Hz, Ar-H), 8.37 (d, 2H, *J* = 7.2 Hz, Ar-H), 8.17(d, 1H, *J* = 7.6 Hz, Ar-H), 7.80 (t, 1H, *J* = 8.0 Hz, Ar-H), 4.11(t, *J* = 6.0 Hz, 2H, 1 × CH_2_), 3.18~3.24 (m, 4H, 2 × CH_2_), 3.11~3.15 (m, 2H, 1 × CH_2_), 2.91 (s, 3H, 1 × CH_3_), 1.85~1.86 (m, 8H, 4 × CH_2_); ^13^C-NMR (D_2_O) δ: 206.26, 166.21, 165.60, 140.89, 134.59, 133.39, 132.11, 131.39, 130.69, 128.68, 128.48, 125.27, 122.21, 49.63, 49.44, 42.27, 41.35, 31.76, 26.64, 26.49, 25.73, 25.35; ESI-MS *m*/*z*: 382.2 [M + H − 2HCl]^+^. Anal. calcd. for C_22_H_29_N_3_O_3_Cl_2_·0.3H_2_O: C 57.47%, H 6.49%, N 9.14%; found: C 57.32%, H 6.61%, N 9.01%.

*2-{3-[4-(3-Aminopropylamino)butylamino]butyl}-6-acetyl-1H-benz[de]isoquinoline-1,3(2H)-dione trihydrochloride* (**5e**). Yield: 84%, white solid. ^1^H-NMR (D_2_O) δ: 8.59 (d, 1H, *J* = 8.4 Hz, Ar-H), 8.28~8.31 (m, 2H, Ar-H), 8.10 (d, 1H, *J* = 8.1 Hz, Ar-H), 7.74 (t, *J* = 10 Hz, 1H, Ar-H), 4.15 (t, *J* = 8.0 Hz, 2H, 1 × CH_2_), 3.15~3.25 (m, 10H, 5 × CH_2_), 2.86 (s, 3H, 1 × CH_3_), 2.12~2.20 (m, 4H, 2 × CH_2_ ), 1.85~1.87 (m, 4H, 2 × CH_2_); ^13^C-NMR (D_2_O) δ: 203.86, 163.91, 163.29, 138.65, 132.25, 131.04, 129.75, 128.80, 128.22, 126.44, 126.14, 122.74, 119.75, 47.04, 45.28, 44.56, 37.49, 36.55, 29.33, 27.63, 26.10, 24.14, 23.75, 22.82; ESI-MS *m*/*z*: 425.3 [M + H − 3HCl]^+^. Anal. calcd. for C_24_H_35_N_4_O_3_Cl_3_·1.5H_2_O: C 51.39%, H 6.83%, N 9.99%; found: C 51.63%, H 6.94%, N 9.72%.

*2-{4-[4-(4-Aminobutylamino)butylamino]butyl}-6-acetyl-1H-benz[de]isoquinoline-1,3(2H)-dione trihydrochloride* (**5f**). Yield: 82%, white solid, ^1^H-NMR (D_2_O) δ: 8.60 (d, 1H, *J* = 8.8 Hz, Ar-H), 8.27 (d, *J* = 8.0 Hz, 2H, Ar-H), 8.11 (d, 1H, *J* = 7.6 Hz, Ar-H), 7.73 (t, *J* = 8.0 Hz, 1H, Ar-H), 4.04 (t, *J* = 6.0 Hz, 2H, 1 × CH_2_), 3.14~3.21 (m, 8H, 4 × CH_2_), 3.10 (t, *J* = 8.0 Hz, 2H, 1 × CH_2_), 2.87 (s, 3H, 1 × CH_3_), 1.81~1.84 (m, 12H, 6 × CH_2_); ^13^C-NMR (D_2_O) δ: 203.62, 163.58, 162.96, 136.29, 132.01, 130.68, 129.54, 128.85, 128.13, 126.27, 125.64, 122.65, 119.58, 47.11, 46.90, 39.73, 38.80, 29.22, 24.09, 23.92, 23.21, 22.88, 22.76, 22.64; ESI-MS *m*/*z*: 453.2 [M + H − 3HCl]^+^. Anal. calcd. for C_26_H_39_N_4_O_3_Cl_3_·1.2H_2_O: C 53.51%, H 7.15%, N 9.60%; found: C 53.21%, H 6.94%, N 9.43%.

### 3.3. General Procedure for the Synthesis of Compounds ***11***

#### 3.3.1. Synthesis of (3-Aminopropyl)-1*H*-benz[*de*]isoquinoline-1,3(2*H*)-dione (**8**)

A mixture of 1,8-naphthalic anhydride (10 mmol) and 1,3-propanediamine (10 mmol) in EtOH was heated at refluxing temperature, and the reaction process was monitored by TLC. After completion, the solvent was removed by evaporation. The resulting solid (**8**) was used in the next step without isolation.

*(3-Aminopropyl)-1H-benz[de]isoquinoline-1,3(2H)-dione* (**8**). Yield: 85%, white solid. ^1^H-NMR (DMSO) δ: 8.44~8.50 (m, 4H, Ar-H), 7.85~7.89 (m, 2H, Ar-H), 4.10 (t, *J* = 8.0 Hz, 2H, 1 × CH_2_), 2.58 (t, *J* = 6.0 Hz, 2H, 1 × CH_2_), 1.68~1.75 (m, 2H, 1 × CH_2_); ESI-MI *m*/*z*: 255.2 [M + 1]^+^.

#### 3.3.2. General Procedure for the Synthesis of **11a**–**b**, **e**–**f**

To a solution of intermediate (**8**) (5 mmol) and anhydrous K_2_CO_3_ (7.5 mmol) in acetonitrile (50 mL), chloroacetyl chloride (6 mmol) in acetonitrile (5 mL) was added at room temperature. The mixture was stirred at room temperature overnight, then compound **9** (1.52 g) was collected by filtration and washed with water (three times with each 10 mL).

A mixture of **9** (1 mmol), anhydrous K_2_CO_3_ (1.5 mmol), a catalytic amount of KI, in DMF (5 mL) and acetonitrile (15 mL) was stirred at room temperature for 0.5 h, then 1 equivalent of Boc protected polyamine **6** was added and the mixture was stirred overnight. After completion, the reaction mixture was concentrated under vacuum to give an oily residue. The residue was then dissolved in CH_2_Cl_2_ (25 mL) and washed with aqueous Na_2_CO_3_ (25 mL). The organic layer was separated, dried with anhydrous Na_2_SO_4_, filtered, and concentrated under vacuum. The residue was purified by chromatography on silica gel using CH_2_Cl_2_/CH_3_OH (100:3 to 100:5) mixture as eluent to yield compounds **10a**–**b**, **e**–**f**. The respective *N*-Boc protected intermediates **10** (1.0 mmol) were dissolved in EtOH (10 mL) and stirred at 0 °C for 10 min. Then 4 M HCl was added dropwise at 0 °C. The reaction mixture was stirred at room temperature overnight. The solution typically gave a white solid as precipitate. The solid was filtered, washed several times with absolute ethanol, and dried under vacuum to give the pure target compounds **11a**–**b**, **e**–**f**.

*2-(3-Aminopropylamino)-N-(3-(1,3-dioxo-1H-benzo[de]isoquinolin-2(3H)-yl)propyl)acetamide dihydrochloride* (**11a**). Yield: 85%, white solid. ^1^H-NMR (D_2_O) δ: 7.75 (t, *J* = 8.0 Hz, 4H, Ar-H), 7.28~7.31 (m, 2H, Ar-H), 3.91 (s, 2H, 1 × CH_2_), 3.69 (t, *J* = 6.0 Hz, 2H, 1 × CH_2_), 3.24 (t, *J* = 6.4 Hz, 2H, 1 × CH_2_), 3.17 (t, *J* = 8.0 Hz, 2H, 1 × CH_2_), 3.07 (t, *J* = 8.0 Hz, 2H, 1 × CH_2_), 2.05~2.13 (m, 2H, 1 × CH_2_), 1.70~1.75 (m, 2H, 1 × CH_2_); ^13^C-NMR (D_2_O) δ: 165.68, 163.82, 134.54, 130.66, 129.61, 126.46, 125.19, 119.01, 48.09, 44.60, 37.94, 37.21, 36.54, 26.57, 23.68; ESI-MI *m*/*z*: 369.3 [M + 1 − 2HCl]^+^. Anal. calcd. for C_20_H_26_Cl_2_N_4_O_3_·0.4H_2_O: C 53.55%, H 6.02%, N 12.49%; found: C 53.65%, H 5.93%, N 12.26%.

*2-(3-(3-Aminopropylamino)propylamino)-N-(3-(1,3-dioxo-1H-benzo[de]isoquinolin-2(3H)-yl)propyl)acetamide trihydrochloride* (**11b**). Yield: 85%, white solid. ^1^H-NMR (D_2_O) δ: 7.82 (t, *J* = 7.6 Hz, 4H, Ar-H), 7.36 (t, *J* = 7.2 Hz, 2H, Ar-H), 3.93 (s, 2H, 1 × CH_2_), 3.74 (t, *J* = 6.0 Hz, 2H, 1 × CH_2_), 3.27 (t, *J* = 6.4 Hz, 2H, 1 × CH_2_), 3.15~3.22 (m, 6H, 3 × CH_2_), 3.08 (t, *J* = 7.6 Hz, 2H, 1 × CH_2_), 2.13~2.17 (m, 2H, 1 × CH_2_), 2.06~2.10 (m, 2H, 1 × CH_2_), 1.73~1.76 (m, 2H, 1 × CH_2_); ^13^C-NMR (D_2_O) δ: 165.62, 163.98, 134.63, 130.76, 129.71, 126.52, 125.32, 119.10, 48.10, 44.71, 44.57, 44.47, 37.96, 37.21, 36.50, 26.57, 23.73, 22.61; ESI-MI *m*/*z*: 426.3 [M + 1 − 3HCl]^+^. Anal. calcd. for C_23_H_34_Cl_3_N_5_O_3_·0.35: C 51.04%, H 6.46%, N 12.94%; found: C 51.04%, H 6.78%, N 12.97%.

*2-(3-(4-(3-Aminopropylamino)butylamino)propylamino)-N-(3-(1,3-dioxo-1H-benzo[de]isoquinolin-2(3H)-yl)propyl)acetamide tetrahydrochloride* (**11e**). Yield: 85%, white solid. ^1^H-NMR (D_2_O) δ: 7.97 (d, *J* = 6.8 Hz, 4H, Ar-H), 7.48 (t, *J* = 7.2 Hz, 2H, Ar-H), 3.93 (s, 2H, 1 × CH_2_), 3.83 (t, *J* = 5.6 Hz, 2H, 1 × CH_2_), 3.28 (t, *J* = 6.4 Hz, 2H, 1 × CH_2_), 3.05~3.21 (m, 12H, 6 × CH_2_), 2.10~2.18 (m, 2H, 1 × CH_2_), 2.04~2.07 (m, 2H, 1 × CH_2_), 1.76~1.81 (m, 6H, 3 × CH_2_); ^13^C-NMR (D_2_O) δ: 165.63, 164.45, 134.63, 130.99, 130.01, 126.69, 125.78, 119.55, 48.10, 47.03, 46.98, 44.52, 44.43, 38.00, 37.26, 36.52, 26.63, 23.72, 22.75, 22.63; ESI-MI *m*/*z*: 497.4 [M + 1 − 4HCl]^+^. Anal. calcd. for C_27_H_44_Cl_4_N_6_O_3_·0.25H_2_O: C 50.12%, H 6.93%, N 12.99%; found: C 50.37%, H 6.65%, N 12.70%.

*2-(4-(4-(4-Aminobutylamino)butylamino)butylamino)-N-(3-(1,3-dioxo-1H-benzo[de]isoquinolin-2(3H)-yl)propyl)acetamide tetrahydrochloride* (**11f**). Yield: 85%, white solid. ^1^H-NMR (D_2_O) δ: 7.71~7.76 (m, *J* = 8.0 Hz, 4H, Ar-H), 7.28 (t, *J* = 8.0 Hz, 2H, Ar-H), 3.82 (s, 2H, 1 × CH_2_), 3.64 (t, *J* = 7.2 Hz, 2H, 1 × CH_2_), 3.19 (t, *J* = 6.4 Hz, 2H, 1 × CH_2_), 2.99~3.06 (m, 10H, 5 × CH_2_), 2.92 (t, *J* = 7.6 Hz, 2H, 1 × CH_2_), 1.65~1.71 (m, 14H, 7 × CH_2_); ^13^C-NMR (D_2_O) δ: 165.80, 164.53, 134.87, 131.06, 130.19, 126.75, 125.96, 119.74, 47.99, 46.91, 46.81, 38.81, 38.06, 38.02, 37.30, 37.28, 26.69, 26.65, 26.64, 23.92, 22.80, 22.76; ESI-MI *m*/*z*: 525.3 [M + 1 − 4HCl]^+^. Anal. calcd. for C_29_H_48_Cl_4_N_6_O_3_·1.05H_2_O: C 50.52%, H 7.32%, N 12.19%; found: C 50.60%, H 7.12%, N 12.42%.

### 3.4. General Procedure for the Synthesis of ***17b***–***d***

#### 3.4.1. Synthesis of Compounds **14a**–**b**

Intermediates **12, 13** were prepared by a modified previously reported procedure [26,27]. Compound **13** (1.13 g, 5 mmol) added portion wise to 1,3-dibromopropane or 1,4-dibromobutane (10 mmol) in acetone (30 mL). The solution was refluxed for 12 h; after cooling and filtration, the solvent was removed under vacuo to give a solid. The latter was recrystallized from ethanol to afford compound **14a**–**b**.

*5-Bromo-2-(3-bromopropyl)isoindoline-1,3-dione* (**14a**), Yield: 70%, ^1^H-NMR (CDCl_3_) δ: 7.97 (d, 1H, *J* = 1.8 Hz, Ar-H), 7.85~7.87 (m, 1H, Ar-H), 7.72 (d, 1H, *J* = 8.0 Hz, Ar-H), 3.71 (t, 2H, *J* = 6.0 Hz, 1 × CH_2_), 3.44 (t, 2H, *J* = 6.4 Hz, 1 × CH_2_), 1.82~1.91 (m, 4H, 1 × CH_2_). ESI-MS *m*/*z*: 345.9 [M + 1]^+^.

*5-Bromo-2-(4-bromobutyl)isoindoline-1,3-dione* (**14b**), Yield: 68%, ^1^H-NMR (CDCl_3_) δ: 7.98 (d, 1H, *J* = 1.2 Hz, Ar-H), 7.84~7.87 (m, 1H, Ar-H), 7.72 (d, 1H, *J* = 7.6 Hz, Ar-H), 3.71 (t, 2H, *J* = 6.8 Hz, 1 × CH_2_), 3.44 (t, 2H, *J* = 6.4 Hz, 1 × CH_2_), 1.81~1.91 (m, 4H, 2 × CH_2_). ESI-MS *m*/*z*: 359.9 [M + 1]^+^.

#### 3.4.2. Synthesis of Compounds **15b**–**d**

Intermediates **14** (1 mmol), Boc protected polyamine (1.2 mmol), anhydrous K_2_CO_3_ (1.5 mmol) were added to the flask in the presence of KI (0.01 mmol) and CH_3_CN (30 mL). The resulting mixture was heated at 45 °C for 12 h, then the solvent was evaporated in vacuo, water was added to the residue, and extracted with CH_2_Cl_2_. The organic layer was dried over Na_2_SO_4_ and evaporated in vacuo. The residue was dissolved in CH_3_OH and a solution of di-*tert*-butyl dicarbonate (1.5 mmol) in methanol (5 mL) was added dropwise at 0 °C then the mixture was stirred for an additional 1 h at 0 °C. The temperature was allowed to gradually rise to room temperature, and the reaction was stirred overnight. The mixture was evaporated in vacuo. The residue was dissolved in CH_2_Cl_2_ and washed with water several times. The organic layer was separated, dried with anhydrous Na_2_SO_4_, filtered, and concentrated. The residue could be purified by flash column chromatography on silica gel.

*5-Bromo-2-{3-[3-(3-butoxycarbonylaminopropylbutoxycarbonylamino)-propylbutoxy-carbonyl-amino]propyl}isoindoline-1,3-dione* (**15b**). Yield: 63%; ^1^H-NMR (CDCl_3_) δ: 7.88 (s, 1H, Ar-H), 7.78 (d, 1H, *J* = 8.0 Hz, Ar-H), 7.63 (d, 1H, *J* = 8.0 Hz), 3.62 (t, 2H, *J* = 6.4 Hz, 1 × CH_2_), 3.03~3.17 (m, 10H, 5 × CH_2_), 1.82 (m, 2H, 1 × CH_2_), 1.35~1.44 (m, 31H, 2 × CH_2_ + 9 × CH_3_); ESI-MS *m*/*z*: 697.3 [M + 1]^+^.

*5-Bromo-2-{3-[4-(3-butoxycarbonylaminopropylbutoxycarbonylamino)-butylbutoxy-carbonyl-amino]propyl}isoindoline-1,3-dione* (**15c**). Yield: 56%; ^1^H-NMR (CDCl_3_) δ: 7.87 (s, 1H, Ar-H), 7.78 (t, 1H, *J* = 6.40 Hz, Ar-H), 7.64 (d, 1H, *J* = 8.0 Hz), 3.65 (t, 2H, *J* = 6.0 Hz, 1 × CH_2_), 3.04~3.18 (m, 10H, 5 × CH_2_), 1.83 (m, 4H, 2 × CH_2_), 1.32~1.40 (m, 31H, 2 × CH_2_ + 9 × CH_3_); ESI-MS *m*/*z*: 711.3 [M + 1]^+^.

*5-Bromo-2-{4-[4-(4-butoxycarbonylaminobutylbutoxycarbonylamino)-butylbutoxy-carbonyl-amino]butyl}isoindoline-1,3-dione* (**15d**). Yield: 60%; ^1^H-NMR (CDCl_3_) δ: 7.88 (s, 1H, Ar-H), 7.77 (t, 1H, *J* = 6.80 Hz, Ar-H), 7.63 (d, 1H, *J* = 6.4 Hz), 3.64 (t, 2H, *J* = 6.6 Hz, 1 × CH_2_), 3.02~3.15 (m, 10H, 5 × CH_2_), 1.31~1.401 (m, 39H, 6 × CH_2_ + 9 × CH_3_); ESI-MS *m*/*z*: 739.3 [M + 1]^+^.

#### 3.4.3. Synthesis of Compounds **17b**–**d**

A 50 mL round bottomed flask equipped with a magnetic stirring bar was charged with pure **15** (2 mmol), toluene (20 mL), phenylboronic acid (2 mmol), K_2_CO_3_ (3 mmol), PdCl_2_ (0.03 mmol) and tetrabutylammonium bromide (0.03 mmol). The mixture was vigorously stirred for 6 h at the boiling temperature. After reaction completion, the reaction mixture was filtered and evaporated to dryness, and the residue was subjected to column chromatography to obtain the intermediates **16b**–**d**. The respective *N*-Boc protected intermediates **16b**–**d** (1.2 mmol) were dissolved in EtOH (20 mL) and stirred at 0 °C for 10 min. Then, 4 M HCl was added dropwise at 0 °C The reaction was stirred at room temperature overnight. The solution typically gave a white solid as a precipitate. The solid was filtered, washed several times with absolute ethanol, and dried under vacuum to give the pure target compounds **17b**–**d**.

*5-Phenyl-2-{3-[3-(3-aminopropylamino)-propylamino]propyl}isoindoline-1,3-dione trihydrochloride* (**17b**). Yield: 78%, ^1^H-NMR (D_2_O) δ: 7.38 (d, 1H, *J* = 8.0 Hz, Ar-H), 7.25~7.33 (m, 2H, Ar-H), 7.21 (t, 3H, *J* = 7.8 Hz, Ar-H), 7.07~7.09 (d, 2H, *J* = 7.6 Hz, Ar-H), 3.68 (t, 2H, *J* = 6.0 Hz, 1 × CH_2_), 3.11~3.22 (m, 10H, 5 × CH_2_), 2.09~2.16 (m, 4H, 2 × CH_2_), 1.90~2.01 (m, 2H, 1 × CH_2_); ^13^C-NMR (D_2_O) δ: 169.33, 168.79, 146.39, 137.55, 132.77, 129.81, 129.17, 128.90, 126.48, 123.86, 120.98, 45.30, 44.69, 44.54, 36.51, 34.81, 24.94, 24.80, 23.72, 22.63; ESI-MS *m*/*z*: 395.3 [M + 1 − 3HCl]^+^. C_23_H_33_Cl_3_N_4_O_2_·0.1H_2_O: C 54.63%, H 6.62%, N 11.08%; found: C 54.59%, H 6.91%, N 10.99%.

*5-Phenyl-2-{3-[4-(3-aminopropylamino)-amino]propyl}isoindoline-1,3-dione trihydrochloride* (**17c**). Yield: 76%; ^1^H-NMR (D_2_O) δ: 7.94~7.96 (m, 1H, Ar-H), 7.90 (d, 1H, *J* = 7.6Hz, Ar-H), 7.80 (s, 1H, Ar-H), 7.73~7.75 (m, 1H, Ar-H), 7.68~7.70 (m, 1H, Ar-H), 7.53~7.56 (m, 1H, *J* = 6.4 Hz, Ar-H), 7.46~7.48 (m, 2H, Ar-H), 3.67~3.74 (m, 2H, 1 × CH_2_), 3.09~3.19 (m, 10H, 5 × CH_2_), 2.02~2.14 (m, 4H, 2 × CH_2_), 1.79~1.80 (m, 4H, 2 × CH_2_); ^13^C-NMR (D_2_O) δ: 169.27, 169.01, 145.93, 137.43, 132.52, 132.16, 131.42, 129.04, 126.53, 123.66, 120.68, 47.04, 45.02, 44.56, 36.56, 34.83, 24.97, 24.79, 23.75, 22.83, 22.77; ESI-MS *m*/*z*: 409.3 [M + 1 − 3HCl]^+^. C_24_H_35_Cl_3_N_4_O_2_·0.1H_2_O: C 55.46%, H 6.83%, N 10.78%; found: C 55.77%, H 6.98%, N 10.75%.

*5-Phenyl-2-{4-[4-(4-aminobutylamino)-butylamino]butyl}isoindoline-1,3-dione trihydrochloride* (**17d**). Yield: 77%, ^1^H-NMR (D_2_O) δ: 7.30~7.31 (m, 1H, Ar-H), 7.18~7.23 (m, 2H, Ar-H), 7.10~7.15 (m, 3H, Ar-H), 8.96~7.01 (m, 2H, Ar-H), 3.29 (t, 2H, *J* = 6.0 Hz, 1 × CH_2_), 2.96~3.06 (m, 10H, 5 × CH_2_), 1.73~1.74 (m, 8H, 4 × CH_2_), 1.60~1.63 (m, 2H, 1 × CH2), 1.45~1.47 (m, 2H, 1 × CH_2_); ^13^C-NMR (D_2_O) δ: 169.69, 169.51, 146.14, 137.29, 132.29, 131.51, 129.10, 129.05, 126.69, 123.61, 120.83, 46.96, 46.89, 46.85, 46.79, 38.75, 36.97, 27.63, 24.87, 23.89, 22.98, 22.79, 22.73; ESI-MS *m*/*z*: 437.3 [M + 1 − 3HCl]^+^. Anal. calcd. for C_26_H_39_Cl_3_N_4_O_2_: C 57.20%, H 7.20%, N 10.26%; found: C 57.35%, H 7.28%, N 10.15%.

### 3.5. Cell Culture

HCT-116 cell line, MDA-MB-231 cell line, K562 cell line and HepG2 cell line were purchased from Shanghai Institute for Biological Science, Chinese Academy of Science (Shanghai, China) and were supplemented with 1 mM glutamine and 10% or 20% (*v*/*v*) FCS (Biological Industries Cromwell, CT, USA). Cells were cultured at 37 °C under a 5% CO_2_ atmosphere.

### 3.6. Cytotoxicity against Cancer Cell Lines

The antiproliferative ability of compounds was evaluated in HCT-116 cells, QSG-7701, MDA-MB-231 cells, K562 and HepG2 cells by the conversion of MTT to a purple formazan precipitate as previously described [39]. Briefly, exponentially growing HCT-116 cells, QSG-7701, MDA-MB-231 cells and HepG2 cells were seeded in 96-well plates (5 × 10^3^ cells to each well) and allowed to attach overnight. After 24 h, the cells were treated with various concentrations (1, 5, 10, 30, and 50 μM) of samples for 48 h, and then MTT (100 μL, 1 mg/mL) was added. After incubation for 4 h at 37 °C, the MTT solution was removed and the crystals of viable cells were dissolved with DMSO (150 μL) in each well; 5000/well exponentially growing K562 were seeded in 96-well plates and treated with indicated concentrations (1, 5, 10, 30, and 50 μM) of samples for 48 h, and then MTT (10 mg/mL, 10 μL) was added. After incubation for 4 h at 37 °C the crystals of viable cells were dissolved overnight with SDS (sodium dodecylsulfonate, 10%, 100 μL) in each well. The absorbance spectra were measured on an ELISA Processor II Microplate Reader (Thermo Scientific, Waltham, MA, USA) at a wavelength of 570 nm. The percentage of cytotoxicity was defined with treated and untreated cell lines. The 50% antitumor activity dose (IC_50_) was defined as the concentration of samples that reduced the absorbance of the treated cells by 50%.

### 3.7. Cellular Apoptotic Evaluation

HepG2 cells were seeded in 96 well plates (6 × 10^3^ cells/well), cultured for 24 h to obtain a confluent monolayer and then treated with various concentrations of tested compounds for 48 h. Cells were incubated with Acridine Orange (50 μM)/ethidium bromide (50 μM) for 30 min, then washed with PBS to remove unbound dyes. Images were obtained on the High Content Screening (HCS, ArrayScan, Thermo Scientific) reader using the Target Activation BioApplication software (Thermo Scientific).

HepG2 cells were seeded in 96 well plates (6 × 10^3^ cells/well), cultured for 24 h to obtain a confluent monolayer, and then treated with various concentrations of tested compounds for 48 h. Cells were incubated with Rh123 (0.25 nM), or DCFH-DA (10 μM), or Lyso-Tracker Red (50 nM ) in the dark at 37 °C for 30 min. for the measurement of mitochondrial membrane potential (MMP), or reactive oxygen species (ROS) or lysosomal mass/pH staining, respectively. After being washed with PBS to remove excess dyes, cells were further stained with the nuclear dye Hoechst 33,342 (10 μM) in the dark at 37 °C for 20 min. Images were acquired on the ArrayScan HCS reader using the Target Activation BioApplication software.

### 3.8. Subcutaneous Xenograft of H22 Cells in Kunming Mice

All animal care and experimentation conformed to the Guide for the Care and Use of Laboratory Animals published by Henan University (HUSOM2016-162). For solid tumor development, 30 male Kunming mice (Laboratory Animal Center of Henan, Zhengzhou, China) aged 5 weeks (weighing 18–22 g) were injected subcutaneously with 2 × 10^6^ H22 cells. On the eighth day after inoculation, the mice were randomly divided into three groups (control, **11e** group, and amonafide group) and then were administered by caudal vein injection **11e** (1 mg/kg) or amonafide (5 mg/kg, as positive control) or physiologic saline for 7 consecutive days. On day 16, the mice were killed by cervical vertebra dislocation, and solid tumors were removed and weighed. The inhibitory rate was calculated as follows: inhibitory rate (%) = [(A − B)/A] × 100, where A was the mean tumor weight of the control group and B was that of the drug treated or positive group.

### 3.9. H22 Cells Lung Metastasis Models

H22 cells (5 × 10^6^ cells per mouse) for Kunming mice were injected intravenous (*i.v.*) through the tail vein for tumor passive metastasis. To ensure all mice bore actively growing lung tumors before the drug treatment, pulmonary metastasis was allowed to develop for 10 days. On day 11, mice were injected intravenously with **11e** (1 mg/kg), amonafide (5 mg/kg, positive, control), and normal saline (negative control) (*n* = 8) for 7 consecutive days. On day 18, mice were killed by cervical vertebra dislocation, and lungs were removed and weighed, and then fixed in Bouin’s fluid. After the lungs were fixed, lung metastases nodus was numbered.

### 3.10. Survival Time in Mice Bearing H22 Cells

For calculation of the survival time, 30 male Kunming mice were inoculated via intraperitoneal (*i.p*.) injection with 1 × 10^6^ H22 cells/mouse on day 0 and were randomly divided into three groups. The treatment with **11e** (1 mg/kg), amonafide (5 mg/kg), or physiologic saline by caudal vein injection was started 24 h after inoculation for 7 consecutive days. The median survival time (MST) for each group was observed, and the antitumor activity of the drug was evaluated by measuring the increase of the lifespan. The extended lifespan rate was calculated as follows: extend rate (%) = (MST treated group/MST control group) × 100.

### 3.11. Systemic Toxicity Evaluation

Mice (8 per group) received **11e** (1 mg/kg) and amonfide (5 mg/kg, *i.v.*) for 7 consecutive days and then were sacrificed. Heart, liver, kidney, lung and spleen were removed and weighed. The organ index was investigated for systemic toxicity evaluation: organ index (%) = (organ weight/body weight) × 100.

## 4. Conclusions

Three series of aromatic imide modified polyamines were synthesized, and their induced growth inhibition of multiple cancer cell lines in vitro was determined using the MTT assay. We found that both the imide scaffold and the polyamine motif affect the biological activities of the designed compounds. One naphthalene imide-polyamine conjugate potently inhibited the growth of multiple cancer cell lines as the reference drug amonafide. The impacts of **11e** on cell morphology, mitochondrial transmembrane potential, and lysosomes indicated that **11e** may impair tumor cells via apoptosis. The present study identified compound **11e** induces apoptosis via a ROS mediated mitochondrial pathway in the preliminary study of cell death profile. Further in vivo experiments on H22 tumor transplant models showed that **11e** prevented the lung cancer metastasis and extended lifespan compared to amonafide. Encouragingly, **11e** inhibited tumor growth and improved body weight more efficiently than amonafide, which strongly corroborated that compound **11e** is worthy of further investigation as a new lead compound for cancer. Collectively, the present report may provide new inspiration for tackling critical issues ranging from naphthalimide/polyamine-based drug design to antitumor drug screening.

## Figures and Tables

**Table 1:**
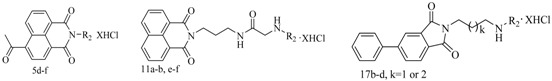
In vitro activity of new compounds against five kinds of tumor cell lines.

Compd.	R_2_	X	k	IC_50_ (μM) ^a^				
				HCT-116	HepG2	K562	MDA-MB-231	QSG-7701
**Amo.**				15.43	6.57	6.31	7.98	55.45
**5d**	-(CH_2_)_4_NH(CH_2_)_4_NH_2_	2		22.92	23.85	31.05	nd ^b^	23.21
**5e**	-(CH_2_)_3_NH(CH_2_)_4_NH(CH_2_)_3_NH_2_	3		16.91	22.07	12.25	nd ^b^	27.65
**5f**	-(CH_2_)_4_NH(CH_2_)_4_NH(CH_2_)_4_NH_2_	3		19.56	30.72	18.81	nd ^b^	21.19
**11a**	-(CH_2_)_3_NH_2_	2		39.85	53.46	33.19	32.38	39.18
**11b**	-(CH_2_)_3_NH(CH_2_)_3_NH_2_	3		>50	>50	28.01	16.75	42.89
**11e**	-(CH_2_)_3_NH(CH_2_)_4_NH(CH_2_)_3_NH_2_	4		5.12	6.33	2.86	11.98	53.85
**11f**	-(CH_2_)_4_NH(CH_2_)_4_NH(CH_2_)_4_NH_2_	4		>50	>50	26.18	57.08	50.64
**17b**	-(CH_2_)_3_NH(CH_2_)_3_NH_2_	3	1	>50	>50	28.17	nd ^b^	>50
**17c**	-(CH_2_)_4_NH(CH_2_)_3_NH_2_	3	1	>50	>50	>50	nd ^b^	>50
**17d**	-(CH_2_)_4_NH(CH_2_)_4_NH_2_	3	2	>50	>50	>50	nd ^b^	>50

^a^ IC_50_ values represent the concentration causing 50% growth inhibition. They were determined by the linear regression method. Each sample is the mean of three independent experiments. ^b^ Not determined. Amo = Amonafide.

**Table 2:** In vivo H22 tumor growth inhibitions by **11e**.

Groups	Dose (mg/kg)	Tumor Weight (g)	Inhibition Rate (%)	No. Dead
Control		2.17 ± 0.22		0/9
Amonafide	5	1.22 ± 0.13	43.79	0/9
**11e**	1	0.63 ± 0.21	70.92	0/9

**Table 3:** In vivo lung metastasis inhibitions by **11e**.

Groups	Dose (mg/kg)	Metastatic Focus (No.)	Inhibition Rate (%)	No. Dead
Control		110.04 ± 15.38		0/8
Amonafide	5	65.25 ± 13.82	40.70	0/8
**11e**	1	40.25 ± 8.15	62.42	0/8

**Table 4:** Physico-chemistry properties and ADMET profile of compound **11e** and amonafide calculated using the Program ACD/Percepta.

Predicted Properties	Compounds	
	11e	Amonafide
MW (g/mol)	496.65	283.33
H-Donors	6	2
H-Acceptor	9	5
Rot. Bonds	18	3
Rings	3	3
Lipinski	1	0
Log P	0.57	1.35
Solubility	1000 mg/mL	21.1 mg/mL
Caco-2	Pe = 0.1 × 10^−6^ cm/s	Pe = 13 × 10^−6^ cm/s
HIA	1%	97%
HLM	0.49	0.46
hERG	0.49	0.39
AMES	0.41	0.57
CNS	−4.81	−3.2

ADMET = Absorption, Distribution Metabolism, Excretion, Toxicity; MW = Molecular weight; HIA = Human intestinal absorption; HLM = Human liver microsomes; hERG = Human ether-a-go-go-related gene; AMES = i.e., probability of positive Ames test; CNS = Central nerous system.

**Table 5:** The meaning of classification score range values for ADMET properties using Program ACD/Percepta.

Classification Scores	Predicted ADMET Properties		
	HLM	hERG	AMES
≤0.33	Stable	Non-inhibitor	Non-mutagenic
>0.33 and ≤0.67	Undefined	Undefined	Undefined
>0.67	Undefined	Inhibitor	Mutagenic
